# Vaginal recurrence of endometrial cancer: MRI characteristics and correlation with patient outcome after salvage radiation therapy

**DOI:** 10.1007/s00261-020-02453-2

**Published:** 2020-04

**Authors:** Aida Steiner, Gabriela Alban, Teresa Cheng, Tina Kapur, Camden Bay, Pierre-Yves McLaughlin, Martin King, Clare Tempany, Larissa J. Lee

**Affiliations:** 1 Department of Radiology, Brigham and Women’s Hospital, 75 Francis Street, L1–050, Boston, MA 02115, USA; 2 Department of Radiology, Turku University Hospital and University of Turku, Kiinamyllynkatu 4-8, 20521 Turku, Finland; 3 Department of Radiation Oncology, Dana-Farber Cancer Institute and Brigham and Women’s Hospital, 75 Francis St, Boston, MA 02115, USA

**Keywords:** Magnetic resonance imaging, Local neoplasm recurrence, Endometrial cancer, Radiotherapy, Survival

## Abstract

**Purpose:**

To evaluate MRI characteristics in vaginal recurrence of endometrial cancer (EC) including tumor volume shrinkage during salvage radiotherapy, and to identify imaging features associated with survival.

**Methods:**

Patients with vaginal recurrence of EC treated with external beam radiotherapy (EBRT) followed by brachytherapy (BT), and with available pelvic MRI at two time points: baseline and/or before BT were retrospectively identified from 2004 to 2017. MRI features including recurrence location and tissue characteristics on T2- and T1-weighted images were evaluated at baseline only. Tumor volumes were measured both at baseline and pre-BT. Survival rates and associations were evaluated by Cox regression and Fisher’s exact test, respectively.

**Results:**

Sixty-two patients with 36 baseline and 50 pre-BT pelvic MRIs were included (24/62 with both MRIs). Vaginal recurrence of EC was most commonly located in the vaginal apex (27/36, 75%). Tumors with a post-contrast enhancing peripheral rim or low T2 signal rim at baseline showed longer recurrence-free survival (RFS) (HR 0.2, 95% CI 0.1–0.9, *P* < 0.05 adjusted for histology; HR 0.2, 95% CI 0.1–0.8, *P* < 0.05, respectively). The median tumor shrinkage at pre-BT was 69% (range 1–99%). Neither absolute tumor volumes nor volume regression at pre-BT were associated with RFS. Lymphovascular space invasion (LVSI) at hysterectomy and adjuvant RT were associated with recurrence involving the distal vagina (both *P* < 0.05).

**Conclusion:**

Vaginal recurrences with rim enhancement at baseline MRI predicted improved RFS, while tumor volume shrinkage at pre-BT did not. Distal vaginal recurrence was more common in patients with LVSI and adjuvant RT at EC diagnosis.

## Introduction

Endometrial cancer (EC) is the most common gynecological cancer in developed countries with an increasing incidence due to the strong association with obesity [1]. Most women are diagnosed with uterine-confined disease [International Federation of Gynecology and Obstetrics (FIGO) stage I] and are managed with total hysterectomy and bilateral salpingo-oophorectomy. Magnetic resonance imaging (MRI) is the imaging modality of choice for pre-operative local staging [2, 3]. Furthermore, MRI enables prognostication by defining the depth of myometrial invasion, which correlates with nodal involvement and survival [4–6]. Despite a favorable prognosis, approximately 10–20% of early stage ECs will relapse, most loco-regionally [7–9]. The recommended treatment for vaginal recurrence is salvage radiation therapy (RT) in the form of external beam RT (EBRT) followed by vaginal brachytherapy (BT), or surgical resection, although there are no randomized prospective studies to guide treatment for these women [10, 11]. Diverse outcomes have been reported after salvage RT, with age, FIGO stage at diagnosis, grade, and histology shown to be prognostic for relapse and survival [10, 12, 13].

Imaging is an essential part of patient evaluation at the time of vaginal recurrence of EC. Whole body computed tomography (CT) and positron emission tomography (PET)/CT are valuable in defining nodal and distant relapse, whereas pelvic MRI is superior to CT in delineating the gynecologic tumor, assessing local invasion, and differentiating recurrence from treatment-related changes [14, 15]. Pelvic MRI also provides high accuracy for target delineation in BT; MRI-guided interstitial BT has shown improved local control compared with CT-guidance in women with vaginal recurrence of EC [16–18]. Furthermore, with the introduction of MRI-guided simulators and linear accelerators, MRI has become an important tool in radiation oncology. Despite increasing utilization of MRI in vaginal recurrence of EC, there are currently no studies describing the different MRI features of these tumors. Furthermore, tumor volumes on MRI and volume shrinkage during RT—known predictors of outcome in cervical cancer—have not been evaluated in vaginal recurrence of EC [19].

The study aims were to characterize qualitative MRI features of the vaginal recurrence both before and during salvage radiation (prior to BT), to evaluate whether MRI appearance of the recurrence relates to primary tumor characteristics, and to investigate the prognostic value of MRI features, tumor volumes, and volume shrinkage. We hypothesize that characterizing these tumors may have profound implications for the radiological assessment and diagnosis, and for guiding radiation oncologists in treatment of vaginal recurrence of EC.

## Materials and methods

### Patients

**Fig. 1:**
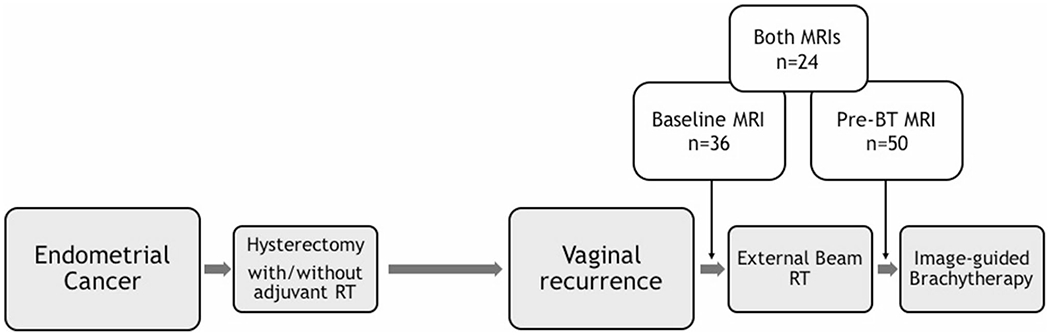
Study algorithm for the timing of baseline MRI and pre-brachytherapy (BT) MRI

This retrospective HIPAA-compliant study was approved by the Institutional Review Board with a waiver of written informed consent. Inclusion criteria were as follows: (1) histologically confirmed first vaginal recurrence of EC post-hysterectomy, (2) treatment with salvage RT including pelvic EBRT and BT at Brigham and Women’s Hospital/Dana-Farber Cancer Institute from April 2004 to January 2018, and (3) available pelvic MRI at the time of recurrence (baseline MRI) and/or prior to BT (pre-BT MRI). The timing of MRIs relative to treatment is illustrated in Fig. 1. Initially, 116 patients were identified from the institutional database. Patients were excluded due to missing MRI (*n* = 48), no EBRT (*n* = 4), MRI not covering the tumor (*n* = 1), and second recurrence (*n* = 1). Finally, a total of 62 patients with 36 baseline and 50 pre-BT MRIs were included. Both MRIs were available in 24 of 62 patients.

### MRI protocol

Pelvic MRIs were obtained at either Brigham and Women’s Hospital/Dana-Farber Cancer Institute (baseline 36%; pre-BT 76%) or elsewhere (baseline 64%; pre-BT 24%). The field strengths used were 1.5 Tesla (*n* = 47), 3.0 Tesla (*n* = 38), and one intraoperative 0.5 Tesla. At a minimum, each study included axial and sagittal T2-weighted (T2WI) and axial T1-weighted images (T1WI). Intravenously administered gadolinium-based contrast agent was used in 35/36 (97%) of baseline and 45/50 (90%) of pre-BT scans. Pre-contrast T1WI was included in all but three pre-BT MRIs. Diffusion weighted (DW) sequence was available in 15/36 (42%) of baseline and 20/50 (40%) of pre-BT scans. Two DWIs were excluded due to artifacts. MRI parameters are detailed in Supplementary Table 1.

### Qualitative MRI features at baseline

MRIs were evaluated in consensus by one abdominal radiology fellow and one senior board certified radiologist with two and 30 years of experience in female pelvic MRI, respectively. Both radiologists were blinded to all clinical and histopathological data. Features at baseline MRI were recorded for each patient and were as follows: (1) morphology (irregular spiculated/lobulated, nodular, round well-defined, i.e., solitary round nodule as opposed to larger nodular mass, or plaque-like along vaginal wall), (2) vaginal location (apex, other), (3) local invasion of neighboring structures (interruption of the normal low T2 signal intensity (SI) of the bladder, urethra, sigmoid, or rectal wall; extension to pelvic sidewall; or hydronephrosis), (4) T1WI SI, (5) T2WI SI relative to pelvic muscles, (6) heterogeneity of SI on T2WI, (7) complete or partial (discontinuous) low SI peripheral rim on T2WI, (8) diffusion restriction (high SI on high *b*-value DWI and low SI on ADC map visually assessed and categorized as none, mild/moderate, or strong), (9) tumor enhancement relative to vaginal mucosa (hypoenhancing or iso/hyperenhancing including heterogeneous enhancement), (10) complete or partial (discontinuous) enhancing peripheral rim, (11) necrosis (non-enhancing, heterogeneous high SI on T2WI), (12) cystic areas (non-enhancing, high SI on T2W similar to bladder), and (13) hemorrhage (bright SI on pre-contrast fat saturated T1WI). MRI features at pre-BT were not recorded.

### Tumor volumes at baseline and pre-BT

Tumor volumes were computed by manually contouring the outer boundary of the lesion on all axial T2WI with visible tumor using 3D Slicer software (https://www.slicer.org/) [20] both at baseline and at pre-BT, and by measuring three maximal perpendicular diameters and using the formula for an ellipsoid volume (*d*_1_ × *d*_2_ × *d*_3_ × *π*/6). Contrast enhanced axial T1WI was used in case of motion artifact on T2WI or superior tumor depiction (*n* = 4). Only diameter-based volume was applied in one patient whose MRI could not be downloaded for contouring. Tumor shrinkage from baseline to pre-BT was calculated as (*V*_baseline_ − *V*_pre-BT_)/*V*_baseline_ × 100%. Contouring was performed by the fellow radiologist in consensus with the senior radiologist or radiation oncologist with expertise in gynecological cancers and MRI-guided BT.

### Statistics

Data characteristics are presented as median with minimum and maximum. Correlations and differences between groups were computed using Spearman’s rank-order correlation and Mann–Whitney *U* tests, respectively. Baseline MRI characteristics were evaluated for their association with histopathology and adjuvant RT using Fisher’s exact test. Rates of recurrence-free survival (RFS) and overall survival (OS) were compared using unadjusted Cox proportional-hazards model. Multivariable Cox regression was employed using rim enhancement and histology as predictors. RFS and OS were defined as the time between histologic diagnosis of vaginal recurrence and second recurrence, or death from any cause, respectively. Patients lost to follow-up and without an event at the time of analysis were censored. Median follow-up was calculated among the patients with censored OS. The optimal prognostic cut-off value for baseline tumor volume was based on Youden’s index derived from a receiver operating characteristic curve predicting RFS at 2-years. Two-tailed *P* values < 0.05 were regarded as statistically significant. Statistical analyses were conducted using IBM SPSS Statistics version 25.0 for Mac (IBM Corp., Armonk, NY, USA). EasyROC web-tool (www.biosoft.hacettepe.edu.tr/easyROC/) was employed for calculating Youden’s index.

## Results

### Patients

Baseline patient characteristics are summarized in Table 1. Mean age was 67 ± 11 years (range 34–88 years). At the time of EC diagnosis and hysterectomy, 84% (52/62) of patients presented with early stage disease (FIGO IA or IB) and 85% (53/62) with endometrioid histology. Seventeen patients (17/62, 27%) had received adjuvant RT including vaginal BT (13/62), pelvic EBRT (2/62), or both (2/62). Median time from EC diagnosis to vaginal recurrence was 22 months (range 2–160 months). At the time of recurrence, patients had restaging evaluation with either CT (44/61) or PET/CT (17/61, information missing for one). All patients underwent salvage RT consisting of pelvic EBRT to a dose of 30.6–54 Gy (median 45 Gy) with an extended field and/or nodal boost when lymph node involvement was suspected, followed by MRI-guided (21/62) or CT-guided (41/62) vaginal BT. Minimum dose delivered to 90% of the high-risk clinical target volume (HR-CTV D90) was 75 Gy (median, range 52–103.4 Gy).

Median time from baseline MRI to EBRT start was 14 days (range 7–68 days), from baseline to pre-BT MRI 60 days (range 31–90 days), and from pre-BT MRI to BT end 10 days (range 2–50 days), respectively. Baseline MRI was performed 7 days after EBRT started in one patient, while the exact date of EBRT start could not be retrieved in another. Both underwent pre-BT MRI 48 and 45 days after baseline, respectively.

### Qualitative MRI features and tumor volumes

**Fig. 2:**
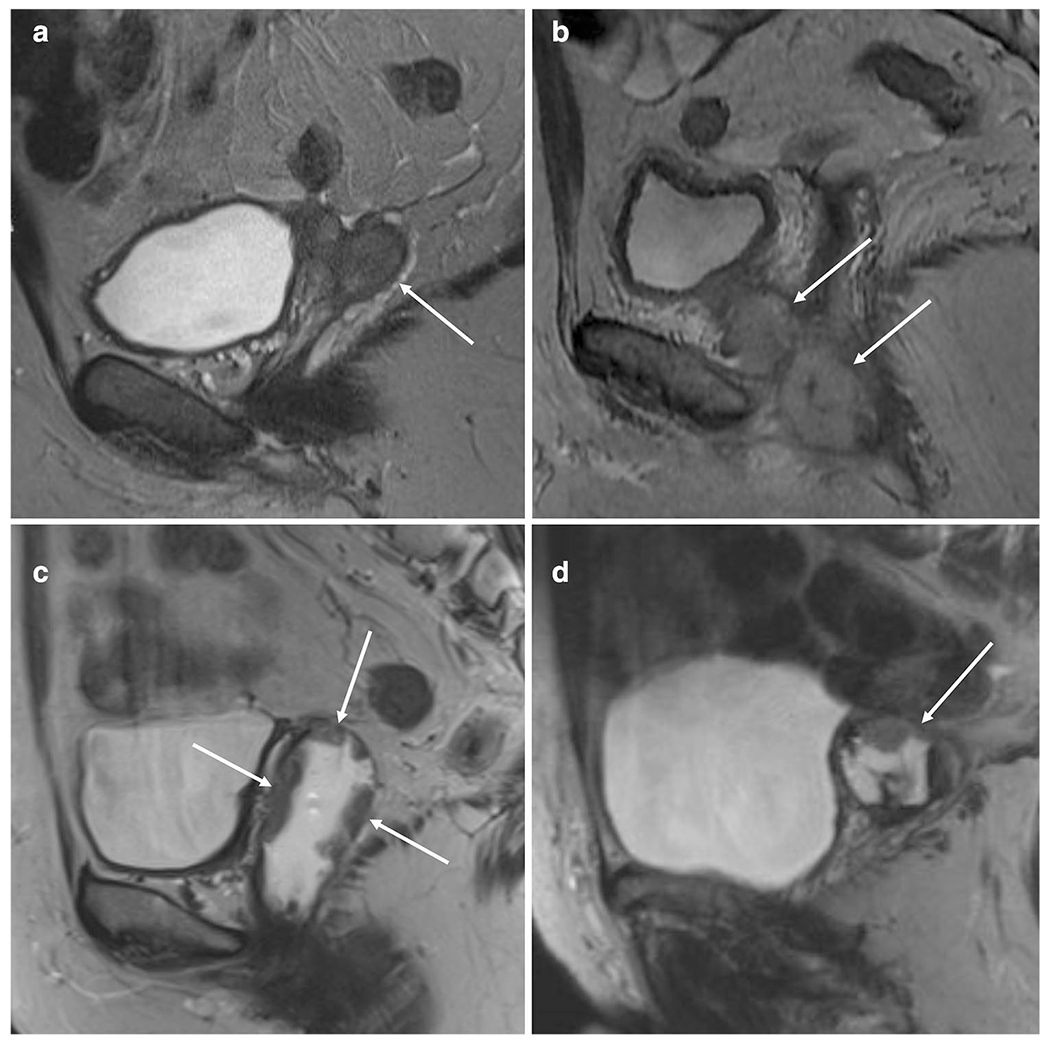
Representative sagittal T2-weighted MR images of vaginal recurrence of endometrial cancer. **a** Typical appearance of an irregular mass in the vaginal apex with intermediate high T2 signal intensity. **b** Vaginal recurrence involving lower third vagina and periurethral space. **c** Atypical appearance of a diffuse plaque-like/papillary tumor growing along the vaginal wall. Vagina is distended with aqueous gel. **d** Complex cyst in the vaginal apex presenting solid mural nodule, septas, hemorrhage, and fluid–fluid level. Histology for each tumor was endometrioid adenocarcinoma

Vaginal apex was the most common location for the vaginal recurrence seen in 27/36 (75%) patients (Table 2). Other locations included the anterior vaginal wall and periurethral space (4/36), lateral wall (3/36), posterior wall (1/36), and diffuse tumor deposits along the vaginal wall (1/36) (Fig. 2). Lower third vagina was involved in 9/36 (25%) patients. The tumor was undetectable at two baseline and three pre-BT MRIs. For these patients, tumor volumes were recorded as 0 cm^3^ and biopsy sites accounted for location. All tumors (solid part) showed hyperintense SI on T2WI and isointense SI on T1WI relative to pelvic muscles. Twenty-nine recurrences out of 33 (88%) were hypoenhancing and 24/33 (73%) showed a hyperenhancing peripheral rim at baseline. Low T2 SI rim was present in 22/34 (65%), 21 out of which co-occurred with the enhancing rim. Strong diffusion restriction was observed whenever DWI was available (14/14). Necrosis was present in 10/33 (30%), cystic areas in 4/33 (12%), and hemorrhage in 4/34 (12%) of the tumors, respectively.

Median tumor volume at baseline was 9.1 cm^3^ (range 0–107 cm^3^) and at pre-BT 2.5 cm^3^ (range 0–108 cm^3^). Corresponding diameter-based tumor volumes were 8.5 cm^3^ (range 0–128 cm^3^) and 2.0 cm^3^ (range 0–124 cm^3^), respectively. Median tumor shrinkage between baseline and pre-BT was 69% (range 1–99%). No correlation was found between baseline tumor volume and volume shrinkage (*r* = 0.07, *P* = 0.74). Non-endometrioid tumors showed less shrinkage (48%) than endometrioid tumors (76%), however, without statistical significance (*P* = 0.15).

### Association of MRI characteristics to histopathology and prior RT

Location of the vaginal recurrence was significantly related to LVSI status and prior RT. Patients who had received adjuvant RT and had positive LVSI in hysterectomy specimen were more likely to present with recurrence involving distal vagina as shown in Table 3. Cystic tumors were associated with grade 3 histology (*P* = 0.008). Necrosis and a cystic component were more frequent with non-endometrioid tumors, however, the association was not significant.

### Prediction of survival based on MRI characteristics and tumor volumes

Median follow-up time was 37 months (range 4–169 months). Two patients were excluded from survival analyses due to lung metastases at diagnosis to minimize the prognostic heterogeneity. One patient with a resected isolated lung nodule at diagnosis was included. Second recurrence after salvage radiation was reported in 23/60 patients (38%) including distant (13/60) or vaginal (5/60) relapse only, vaginal and distant (4/60), and vaginal relapse with pelvic nodes (1/60), respectively. Seventeen out of 60 patients (28%) had died. The cause of death was not recorded as the analysis included only overall survival and not disease-specific mortality. Non-endometrioid histology was a powerful predictor for poor RFS and OS (Supplementary Table 2). Grade 3 at hysterectomy predicted poor OS, but not RFS. Use of an interstitial applicator at BT showed improved RFS, although statistically not significant, compared with intracavitary approach. Optimal dose delivered at BT (HR-CTV D90 ≥ 75 Gy) [10], age, FIGO stage, LVSI, adjuvant RT, time to recurrence, or site of recurrence were not prognostic (data not shown).

**Fig. 3:**
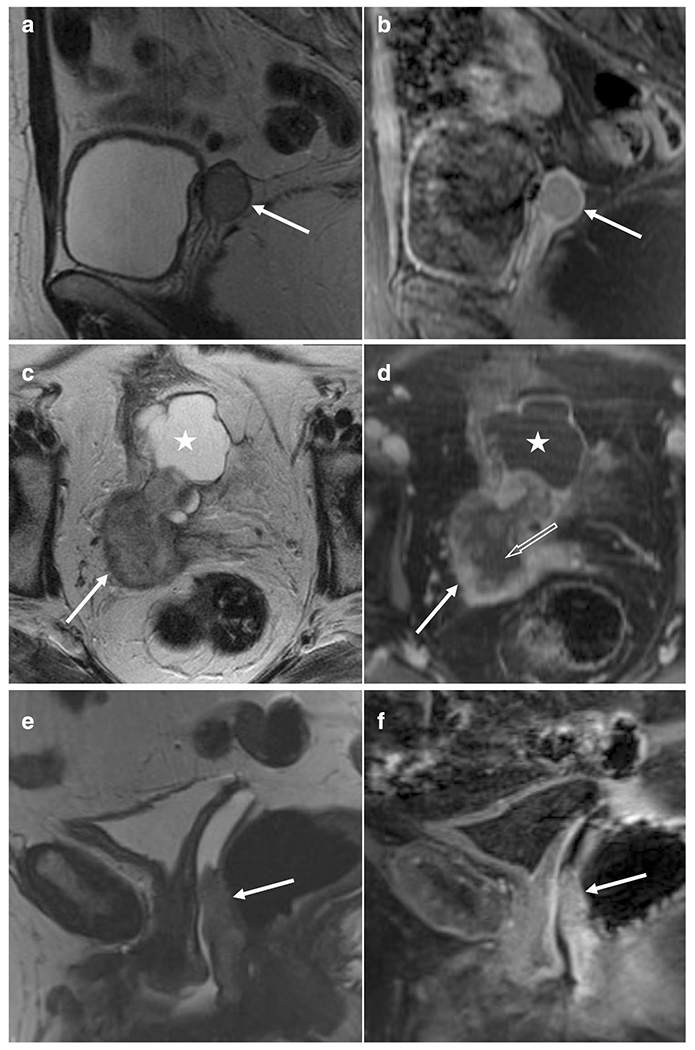
T2-weighted (left column) and contrast-enhanced fat-saturated T1-weighted (right column) MR images representing three separate tumors with or without an enhancing peripheral rim. **a**, **b** Round well-defined recurrence in the vaginal apex showing low signal peripheral rim on T2WI (**a**) that is enhancing (**b**). Histology: endometrioid adenocarcinoma. **c**, **d** Large nodular tumor with cystic (white star) and necrotic (open white arrow) component showing low signal peripheral rim on axial T2WI **c** that was enhancing (**d**). Histology: mixed clear cell adenocarcinoma. **e**, **f** Atypical location in the posterior lower vaginal wall. The tumor shows no low T2 signal rim (**e**) and no rim enhancement (**f**). Histology: serous carcinoma

Table 4 presents prognostication using MRI characteristics (baseline) and tumor volumes (baseline and pre-BT). The presence of an enhancing or low T2 SI peripheral rim (Fig. 3) were associated with lower risk for a second recurrence. Furthermore, enhancing rim independently predicted improved RFS after adjusting for histology (Table 4). Neither enhancing or low T2 SI rim showed any association with histology, grade, or LVSI status (all *P* > 0.60). Regarding prior adjuvant RT, enhancing rim was more frequent in recurrences with history of RT (10/10, 100%) as opposed to recurrences without prior RT (14/23, 61%; *P* = 0.032). Tumors with low T2 SI rim were significantly bigger than tumors without the rim (*P* = 0.013). Plaque-like morphology associated with poor RFS; however, only three patients were identified with this morphology. Tumor location, local invasion, T2 heterogeneity, cystic areas, or hemorrhage were not prognostic (data not shown). Tumor enhancement and round morphology were not included in analyses due to the lack of events in one of the groups.

Tumor volume at baseline MRI (segmented) predicted OS but not RFS (Table 4). A cut-off value was obtained that showed a baseline volume of ≥ 24.7 cm^3^ (6/36) predicted poor OS (HR 6.88, 95% CI 1.09–43.4, *P* = 0.040). Using diameter-based “ellipsoid” tumor volume at baseline MRI resulted in similar outcome predicting OS (HR 1.03, 95% CI 1.01–1.06, *P* = 0.010). No statistically significant association was found between tumor volume at pre-BT MRI or volume shrinkage and survival in this cohort.

## Discussion

This study represents the first systematic evaluation and prognostication of MRI features in EC vaginal recurrence treated with salvage RT. We found that tumors with an enhancing or low T2 SI peripheral rim at baseline had improved RFS rates. Enhancing rim independently predicted RFS after adjusting for histology, a powerful predictor for survival in these patients. An unexpected finding was that tumor volume shrinkage during salvage RT was not prognostic, although this analysis included only 24 patients. Women with LVSI at hysterectomy and those who received adjuvant RT were more likely to present with recurrence involving the distal vagina.

To date, MRI appearance of EC vaginal recurrence has only been described in case-based reviews showing a mass of intermediate T2 SI in the vaginal vault [21, 22]. We report additional atypical appearances including complex cystic, diffuse plaque-like, and distal vaginal lesions. Recognizing the infrequent features of these tumors is crucial for accurate diagnosis and treatment planning.

Enhancing and low T2 SI peripheral rim both predicted improved RFS, even though the tumors with the rim were more bulky. The peripheral rim has not previously been described in vaginal recurrences. In contrast, low T2 SI rim is a characteristic in uterine cervical cancers with expansive growth pattern and is considered to be compressed parenchyma around the tumor [23–25]. Yoshida et al. analyzed cervical MRIs of 452 patients and found that expansive tumors with thin peripheral lines of low T2 SI responded more favorably to chemoradiation than cervical cancers with infiltrative pattern [25]. We did not have a histological specimen for comparison but hypothesize that the rim may be related to compressed parenchyma and/or inflammatory response that restricts infiltrative growth that results in improved RFS. The rim showed early rather than late enhancement suggesting well-vascularized tissue and arguing against merely fibrotic tissue that usually presents with late enhancement.

In cervical cancer, tumor volume regression at MRI during chemoradiotherapy has greater prognostic value than either pre- or post-treatment tumor volumes [26]. Volume shrinkage < 90% at the time of brachytherapy has been associated with poor outcome [19]. There are no studies evaluating volume regression by MRI in vaginal recurrence of EC treated with salvage RT. Some studies have shown association between the size of recurrence (clinical examination/CT) and patient outcome [27–30], while others have not [13, 31]. We found that vaginal recurrences larger than 24.7 cm^3^ at baseline predicted poor OS, but not RFS. No association was found between pre-BT tumor volume or shrinkage and survival. This may be related to improved tumor control with interstitial image-guided BT which is used for larger tumors. However, it cannot be excluded that the variation in pre-BT MRI timepoints affected volumetrics, given the dynamic process of tumor regression during RT.

Patients with positive LVSI at hysterectomy and history of adjuvant RT were more likely to present with recurrence involving the lower vagina. Although the planning target volumes of the adjuvant RT regimens were not analyzed, we assume that the lower vagina was not included or it received lower dose than the proximal part, as recommended by guidelines [32, 33]. The presence of LVSI may provide an additional explanation for the lower vagina involvement. LVSI is defined as the presence of tumor cells in lymphovascular spaces outside the immediate tumor border, and has been reported as an important risk factor for nodal and distant metastases in patients with EC [34–36]. Although no association between LVSI and vaginal recurrence was found in a large cohort of patients from two prospective trials, the vaginal tumors were not defined by location, and therefore, the association between LVSI and lower vaginal relapse remains to be elucidated [36].

Our study has several limitations. First, it is a retrospective analysis of patients treated over a 13-year period during which there has been evolution of the RT regimens, MRI protocols, and adjuvant treatments. Variations in MRI protocols may also have affected the contrast of the image. Second, selection bias may have been present since only patients treated at a specialized academic center for gynecological BT were included. MRI may have been requested in more complex cases. Third, MRIs were evaluated in consensus by a fellow and a senior radiologist preventing evaluation of interobserver variability. Finally, differentiating residual disease from RT-related change was challenging in select cases due to the well-known overlap in imaging features [14]. All DWI, CE T1WI, and follow-up imaging findings were taken into consideration when available.

In conclusion, vaginal recurrences with an enhancing or low T2 SI peripheral rim at baseline MRI were associated with lower risk for a second recurrence after salvage radiation. Tumor volumes at baseline or pre-BT, or the volume regression did not predict second recurrence, which may be related to improved outcomes using interstitial image-guided BT. Patients with LVSI at hysterectomy and those who received adjuvant RT were more likely to present with recurrence involving the distal vagina, with potential implications for adjuvant treatment and follow-up of patients. Our study represents to date the largest series examining MRI features and their prognostic implications in vaginal recurrence of EC.

## Supplementary Material

Suppl. Table 2

Suppl. Table 1

## Figures and Tables

**Table 1:** Clinical characteristics

	Patients, *n* (%)
Histology (hysterectomy)	
Endometrioid carcinoma	53/62 (85%)
Non-endometrioid carcinoma	9/62 (15%)
FIGO grade^a^	
G1	29/62 (47%)
G2	19/62 (31%)
G3	14/62 (22%)
FIGO stage at diagnosis^a^	
IA	39/62 (63%)
IB	13/62 (21%)
II	4/62 (6%)
III	5/62 (8%)
IVB	1/62 (2%)
LVSI	
No	39/62 (63%)
Yes	15/62 (24%)
Missing	8/62 (13%)
Adjuvant therapy after hysterectomy	
None	41/62 (66%)
RT (EBRT and/or BT)	14/62 (23%)
Chemotherapy	4/62 (6%)
RT + chemotherapy	3/62 (5%)
Site of 1st recurrence after hysterectomy	
Vaginal only	51/62 (82%)
Vaginal + pelvic lymph nodes	8/62 (13%)
Vaginal + lung metastasis	3/62 (5%)
Type of BT	
Interstitial	42/62 (68%)
Intracavitary	20/62 (32%)
Chemotherapy at 1st recurrence	
No	39/62 (63%)
Yes	23/62 (37%)

*FIGO* International Federation of Gynecology and Obstetrics, *LVSI* lymphovascular space invasion, *RT* radiotherapy, *EBRT* external beam RT, *BT* brachytherapy

a According to 2009 FIGO classification

**Table 2:** Frequency of MRI characteristics in vaginal recurrence of endometrial cancer at baseline MRI

MRI features	Baseline MRI *N* (%)
Tumor morphology and location	
Morphology	
Irregular lobulated/spiculated	15/34 (44%)
Nodular	13/34 (38%)
Round well-defined	3/34 (9%)
Plaque-like along vaginal wall	3/34 (9%)
Vaginal location	
Vaginal apex	27/36 (75%)
Other	9/36 (25%)
Local invasion of adjacent organs^a^	
No	20/36 (56%)
Yes	16/36 (44%)
Tissue characteristics	
Heterogeneity of SI on T2WI	
Homogeneous	19/34 (56%)
Heterogeneous	15/34 (44%)
Low SI peripheral rim on T2WI	
No	12/34 (35%)
Yes	22/34 (65%)
Tumor enhancement^b^	
Hypoenhancing	29/33 (88%)
Iso/hyperenhancing	4/33 (12%)
Enhancing peripheral rim	
No	9/33 (27%)
Yes	24/33 (73%)
Necrosis	
No	23/33 (70%)
Yes	10/33 (30%)
Cystic areas	
No	29/33 (88%)
Yes	4/33 (12%)

*SI* signal intensity, *T2WI* T2-weighted images

a Invading bladder, urethra, sigmoid, or rectum, extending to pelvic sidewall, or presence of hydronephrosis

b Relative to vaginal mucosa

**Table 3:** Association of MRI characteristics at baseline to histology, grade, LVSI, and adjuvant RT post-hysterectomy

	Involving lower vagina			Necrosis			Cystic areas		
	No	Yes	*P*	No	Yes	*P*	No	Yes	*P*
Histology									
Endometrioid	24/32 (75%)	8/32 (25%)	1.000	22/29 (76%)	7/29 (24%)	0.073	27/29 (93%)	2/29 (7%)	0.062
Non-endometrioid	3/4 (75%)	1/4 (25%)		1/4 (25%)	3/4 (75%)		2/4 (50%)	2/4 (50%)	
Grade									
G1	15/20 (75%)	5/20 (25%)	0.881	14/18 (78%)	4/18 (22%)	0.136	18/18 (100%)	0/18 (0%)	0.008 **
G2	8/10 (80%)	2/10 (20%)		7/9 (78%)	2/9 (22%)		8/9 (89%)	1/9 (11%)	
G3	4/6 (67%)	2/6 (33%)		2/6 (33%)	4/6 (67%)		3/6 (50%)	3/6 (50%)	
LVSI									
No	19/23 (83%)	4/23 (17%)	0.027 *	16/21 (76%)	5/21 (24%)	0.209	20/21 (95%)	1/21 (5%)	0.483
Yes	3/8 (37%)	5/8 (63%)		4/8 (50%)	4/8 (50%)		7/8 (88%)	1/8 (12%)	
Adjuvant RT									
No	22/25 (88%)	3/25 (12%)	0.012 *	18/23 (78%)	5/23 (22%)	0.215	20/23 (87%)	3/23 (13%)	1.000
Yes	5/11 (45%)	6/11 (55%)		5/10 (50%)	5/10 (50%)		9/10 (90%)	1/10 (10%)	

*LVSI* lymphovascular space invasion, *RT* radiation therapy

* *P* < 0.05;

** *P* < 0.01

**Table 4:** Unadjusted and multivariable analysis with Cox proportional-hazards model for survival considering tumor volumes on MRI and MRI characteristics at baseline

	Recurrence-free Survival			Overall Survival		
	HR	95% CI	*P*	HR	95% CI	*P*
Unadjusted analysis						
MRI tumor volume (per cm^3^)						
Baseline	1.00	0.98–1.03	0.994	1.04	1.01–1.06	0.014*
Pre-BT	1.00	0.98–1.02	0.950	1.01	0.99–1.02	0.416
Volume shrinkage	0.99	0.97–1.01	0.409	1.00	0.97–1.03	0.762
MRI characteristics baseline						
Morphology^a^						
Irregular lobulated/spiculated	1			1		
Nodular	0.79	0.18–3.53	0.753	4.36	0.49–39.09	0.188
Plaque-like along vaginal wall	26.95	2.50–290.09	0.007**	9.90	0.80–122.84	0.074
Involving lower 1/3 vagina						
No	1			1		
Yes	1.49	0.31–7.12	0.618	0.82	0.10–6.81	0.857
Low T2 SI peripheral rim						
No	1			1		
Yes	0.27	0.08–0.95	0.042*	0.79	0.18–3.52	0.752
Enhancing peripheral rim						
No	1			1		
Yes	0.27	0.08–0.96	0.043*	1.14	0.22–5.92	0.878
Necrosis						
No	1			1		
Yes	1.24	0.32–4.84	0.754	3.77	0.83–17.15	0.086
Multivariable analysis						
Histology						
Endometrioid	1			1		
Non-endometrioid	5.45	1.32–22.48	0.019*	8.26	1.84–37.18	0.006**
Enhancing peripheral rim						
No	1			1		
Yes	0.25	0.07–0.91	0.035*	1.07	0.20–5.72	0.935

*SI* signal intensity

a No events reported for patients with round well-defined tumors

* *P* < 0.05,

** *P* < 0.01

## Abbreviations

EC: Endometrial cancer
RT: Radiation therapy
EBRT: External beam RT
BT: Brachytherapy
RFS: Recurrence-free survival
OS: Overall survival
CI: Confidence interval
HR: Hazard ratio
LVSI: Lymphovascular space invasion
SI: Signal intensity
