# Supplementary material for: Vaginal recurrence of endometrial cancer: MRI characteristics and correlation with patient outcome after salvage radiation therapy
Source: Abdom Radiol (NY). Author manuscript; Available in PMC 2020 Nov 5. (PMC7643338; doi:10.1007/s00261-020-02453-2)
Supplement: Suppl. Table 2 [file NIHMS1640082-supplement-Suppl__Table_2.docx]

**Supplementary Table 2** Unadjusted analysis with Cox proportional-hazards model for survival, considering clinical characteristics

| Variable | Recurrence-free Survival | | | Overall Survival | | |
| --- | --- | --- | --- | --- | --- | --- |
|  | HR | 95% CI | *P* | HR | 95% CI | *P* |
| Age | 1.01 | 0.97-1.05 | 0.582 | 1.04 | 0.99-1.10 | 0.121 |
| Histology |  |  |  |  |  |  |
| Endometrioid | 1 |  |  | 1 |  |  |
| Non-endometrioid | 3.76 | 1.54-9.19 | 0.004****** | 4.85 | 1.86-12.65 | 0.001****** |
| FIGO Grade |  |  |  |  |  |  |
| G1 | 1 |  |  | 1 |  |  |
| G2 | 0.81 | 0.29-2.23 | 0.681 | 1.43 | 0.38-5.44 | 0.597 |
| G3 | 1.57 | 0.60-4.16 | 0.360 | 3.39 | 1.01-11.35 | 0.048***** |
| Type of BT |  |  |  |  |  |  |
| Intracavitary | 1 |  |  | 1 |  |  |
| Interstitial | 0.46 | 0.20-1.04 | 0.060 | 0.51 | 0.19-1.34 | 0.172 |
| HR-CTV D90 |  |  |  |  |  |  |
| ≥ 75 Gy | 1 |  |  | 1 |  |  |
| < 75 Gy | 0.62 | 0.26-1.49 | 0.286 | 0.72 | 0.25-2.13 | 0.555 |

HR, hazard ratio; CI, confidence interval; FIGO, International Federation of Gynecology and Obstetrics; BT, brachytherapy; HR-CTV D90, minimum dose delivered to 90% of the high risk clinical target volume; Gy, gray; **P*<0.05; ***P*<0.01
