# Supplementary material for: Vaginal recurrence of endometrial cancer: MRI characteristics and correlation with patient outcome after salvage radiation therapy
Source: Abdom Radiol (NY). Author manuscript; Available in PMC 2020 Nov 5. (PMC7643338; doi:10.1007/s00261-020-02453-2)
Supplement: Suppl. Table 1 [file NIHMS1640082-supplement-Suppl__Table_1.docx]

**Supplementary Table 1** The range of MRI acquisition parameters in 1.5 Tesla (T) and 3 T MRI scanners

| MRI Sequence | Slice thickness (mm) | Interslice gap (mm) | Repetition time (ms) | Echo time (ms) | Bandwith (kHz) | Flip angle | Matrix | Highest b value |
| --- | --- | --- | --- | --- | --- | --- | --- | --- |
| 1.5 T scanner, n=47 |  |  |  |  |  |  |  |  |
| T2w SE ^a^ | 3–8 | 0.4–1.8 | 3064–8860 | 77–120 | 81–260 | 90–180 | 256–508 × 192–504 | – |
| T1w contrast enhanced fs 2D GRE or SE | 4–5.5 | 0.8–1.5 | 170–612 | 4.2–21 | 70–244 | 75–90 | 256–288 × 128–224 | – |
| T1w contrast enhanced fs 3D GRE | 1–5 | 0 | 3.7–10.9 | 1.8–7.1 | 130–599 | 10–15 | 124–320 × 125–256 | – |
| EP-DWI | 3.6–8 | 0–1.8 | 1569–9100 | 64–146 | 1008–1953 | 90 | 128–192 × 95–192 | 800–1000 |
| 3 T scanner, n=38 |  |  |  |  |  |  |  |  |
| T2w SE | 3–5 | 0.0–1.1 | 3000–7094 | 82–129 | 163–300 | 90–160 | 320–384 × 205–384 | – |
| T1w contrast enhanced fs 3D GRE | 1–5 | 0 | 3.2–7.2 | 1.2–3.8 | 244–755 | 8–20 | 224–384 × 109–346 | – |
| EP-DWI ^b^ | 3–6 | 0.5–2 | 1800–13000 | 52–106 | 723–2441 | 90–180 | 120–192 × 78–160 | 500–1000 |

T2w, T2-weighted; SE, spin echo; T1w, T1-weighted; fs, fat-saturated; 2D, 2-dimensional acquisition; GRE, gradient echo; 3D, 3-dimensional acquisition; EP-DWI, echo-planar diffusion-weighted imaging. ^a^ All but five without fat saturation; ^b^ Including read-out segmented EP (RESOLVE)
